# Adaptive deep learning model with anatomical priors for 3D segmentation of fine hepatic vasculature on CT

**DOI:** 10.1093/radadv/umag038

**Published:** 2026-11-03

**Authors:** Zixuan Zhao, Kalyani Vyapari, Kyunghyun Sung, David S Lu, Grace H Kim, Matthew S Brown

**Affiliations:** Physics and Biology in Medicine Graduate Program, David Geffen School of Medicine at UCLA, Los Angeles, CA 90065, United States; Center for Computer Vision and Imaging Biomarkers, Department of Radiological Sciences, David Geffen School of Medicine at UCLA, Los Angeles, CA 90025, United States; Center for Computer Vision and Imaging Biomarkers, Department of Radiological Sciences, David Geffen School of Medicine at UCLA, Los Angeles, CA 90025, United States; Physics and Biology in Medicine Graduate Program, David Geffen School of Medicine at UCLA, Los Angeles, CA 90065, United States; Center for Computer Vision and Imaging Biomarkers, Department of Radiological Sciences, David Geffen School of Medicine at UCLA, Los Angeles, CA 90025, United States; Center for Computer Vision and Imaging Biomarkers, Department of Radiological Sciences, David Geffen School of Medicine at UCLA, Los Angeles, CA 90025, United States; Physics and Biology in Medicine Graduate Program, David Geffen School of Medicine at UCLA, Los Angeles, CA 90065, United States; Center for Computer Vision and Imaging Biomarkers, Department of Radiological Sciences, David Geffen School of Medicine at UCLA, Los Angeles, CA 90025, United States; Physics and Biology in Medicine Graduate Program, David Geffen School of Medicine at UCLA, Los Angeles, CA 90065, United States; Center for Computer Vision and Imaging Biomarkers, Department of Radiological Sciences, David Geffen School of Medicine at UCLA, Los Angeles, CA 90025, United States

**Keywords:** hepatic vessel segmentation, deep learning, artificial intelligence, multimodal fusion, CT

## Abstract

**Background:**

Accurate segmentation of fine hepatic vessels on CT remains challenging because peripheral branches are small and low contrast.

**Purpose:**

To develop and test PriM Fusion Net, a deep learning model that adaptively integrates an optimized anatomical prior to improve 3D CT segmentation of portal and hepatic veins, emphasizing fine peripheral branches for preoperative 3D modeling.

**Materials and methods:**

This retrospective study used the public Medical Segmentation Decathlon (MSD) Task08-HepaticVessel dataset of 303 abdominal CT volumes. PriM Fusion Net uses a dual-encoder architecture: a Swin Transformer processes CT images and a parallel convolutional pathway processes a dataset-optimized Frangi vesselness prior. Gated multimodal units act as spatially varying voxel-wise gates that fuse the 2 feature streams at multiple scales. A rule-based SimpleMind post-processing module refined anatomical plausibility. Performance was evaluated with 5-fold cross-validation and zero-shot external inference on 3DIRCADb (3D Image Reconstruction for Comparison of Algorithm Database).

**Results:**

PriM Fusion Net achieved a mean Dice similarity coefficient (DSC) of 77.68% (95% confidence interval [CI]: 77.11, 78.25) and 95th percentile Hausdorff distance (HD95) of 11.55 mm (95% CI, 9.94, 13.16), outperforming all baselines (all adjusted *P* < .02). For vessels with radius ≤ 1.5 mm, DSC improved by 13.5% versus SwinUNETR (59.2% [95% CI, 54.48, 63.92] vs 45.7% [95% CI, 40.49, 50.91]). In ablation, replacing rigid concatenation with gated fusion improved DSC from 75.86% (95% CI, 75.31, 76.41) to 77.65% (95% CI, 76.92, 78.38), and SimpleMind post-processing reduced HD95 from 14.98 mm (95% CI, 10.91, 19.05) to 11.55 mm (95% CI, 9.94, 13.16). On 3DIRCADb, zero-shot inference yielded DSC of 68.42% (95% CI, 65.82, 71.02) and HD95 of 11.78 mm (95% CI, 8.50, 15.06).

**Conclusion:**

Adaptive deep fusion of an optimized anatomical prior improved CT segmentation of portal and hepatic veins, especially fine peripheral branches, supporting further evaluation in 3D vascular modeling workflows for liver surgery and intervention.

**Abbreviations** 

CI = confidence interval, CNN = convolutional neural network, DL = deep learning, DSC = Dice similarity coefficient, FLOP = floating-point operation, GMU = gated multimodal unit, HD95 = 95th percentile Hausdorff distance

**Summary** 

The proposed deep learning model adaptively fuses CT features with an anatomical prior to improve segmentation of portal and hepatic veins, with the largest gains seen in fine peripheral branches.

**Key Results** 

- PriM Fusion Net improved 5-fold cross-validation performance versus all baselines (DSC, 77.68% [95% CI, 77.11, 78.25]; HD95, 11.55 mm [95% CI, 9.94, 13.16]; all adjusted *P* < .02).
- For vessels ≤ 1.5 mm, DSC improved from 45.7% to 59.2% and sensitivity from 42.3% to 56.4% versus SwinUNETR (all adjusted *P* < .02).
- External zero-shot testing on 3DIRCADb also favored PriM Fusion Net over SwinUNETR, supporting robustness under domain shift.

## Introduction

Surgical intervention, including resection and transplantation, remains the first-line curative therapy for patients with primary and metastatic liver malignancies.^1^^,^^2^ The success of these complex procedures depends on a detailed 3D understanding of the hepatic vascular anatomy,^3^ for which high-fidelity 3D models from CT scans are invaluable preoperative tools. Large-scale studies demonstrate that their usage is associated with improved perioperative outcomes, such as shorter operation times and lower morbidity.^4^ Because portal and hepatic venous anatomy defines functional liver territories, segmentation errors can propagate into downstream planning tasks such as future liver remnant estimation and segment-oriented resection design.^5^^,^^6^

However, the generation of these essential vascular models is not yet a routine, automated part of the clinical workflow. In practice, vessel segmentation often depends on labor-intensive manual or semi-automated delineation by expert readers, which limits scalability and introduces inter-observer variability.^7^ This automation gap is critical because the vascular trees serve as the primary anatomical landmarks for defining the liver’s functionally independent Couinaud segments (Figure 1A). Missing peripheral tributaries may reduce the fidelity of these functional maps and can obscure variant anatomy that matters during parenchymal-sparing surgery, venous reconstruction, and selective embolization planning.^8^

**Figure 1:**
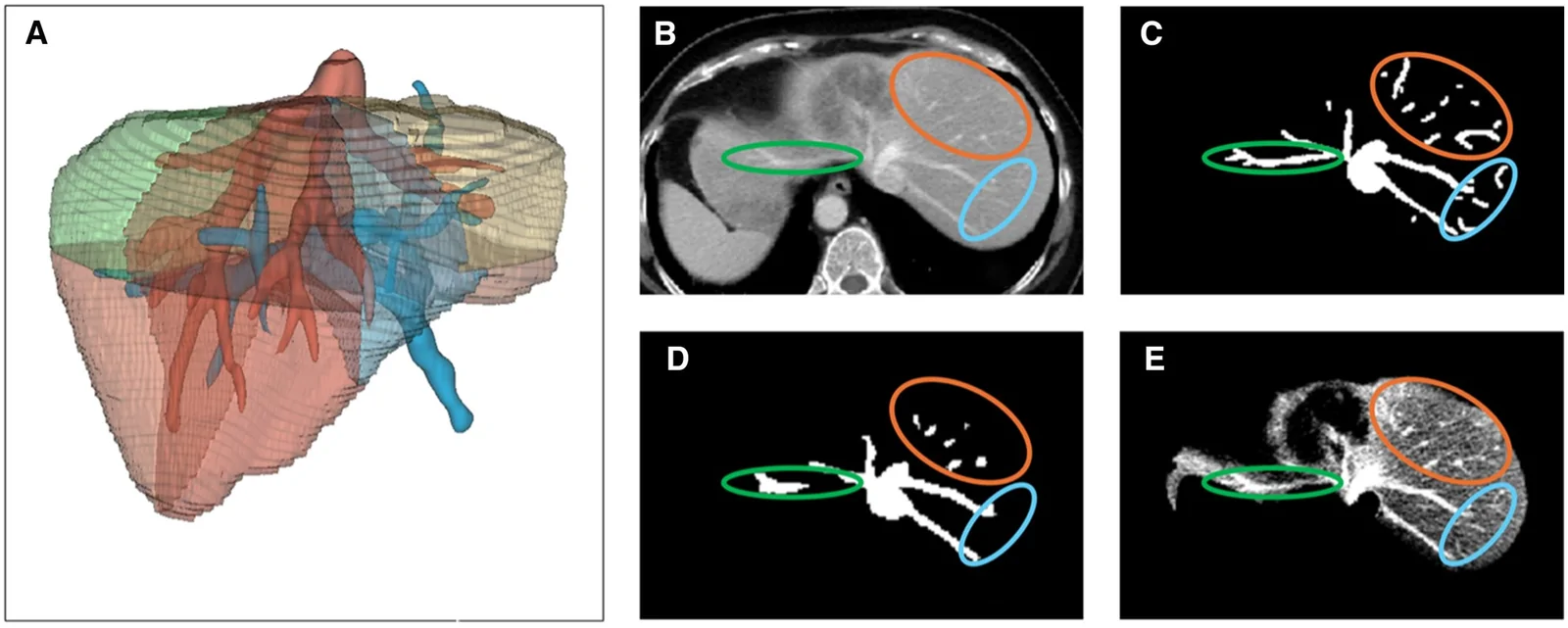
Motivation for our work. (A) An example showing 3D visualization of the hepatic vasculature relative to the Couinaud segments. (B) A representative axial CT slice shows 3 regions of interest (ROIs) where fine vessels are difficult to distinguish from the surrounding parenchyma. Scale bar = 5 cm. (C) The ground truth segmentation. (D) The output from the nnU-Net model was unable to detect these low-contrast vessels. (E) The vesselness map computed via an optimized Frangi filter, which reasonably delineates these small vessels within the ROIs. Modality: contrast‑enhanced CT; plane: axial for panels (B-E).

Recent deep learning (DL) developments such as U-Net^9^ and Transformer-based models^10^ have shown promise in capturing global context but struggle to reliably segment the fine, low-contrast vessels that are critical for surgical planning. As shown in Figure 1, DL architectures like nnU-Net^11^ (Figure 1D) struggle to delineate the fine, low-contrast vessels (Figure 1B) that are clearly part of the complete vascular tree (Figure 1C). This motivates the integration of anatomical priors. For instance, a vesselness filter can enhance these tubular structures, revealing details that the DL network was less sensitive to (Figure 1E). Yet, the common approach of rigid “early fusion”—concatenating the prior as an input channel—has shown limited success. Garret et al.^12^ explored this with multiple vesselness filters, finding that while it improved small vessel detection, it degraded performance for larger vessels. Adaptive (Deep) Fusion aims to dynamically moderate the influence of the prior. This can be achieved through attention mechanisms that re-weight feature maps after they have been combined.^13^^,^^14^

An alternative, more direct approach is to use a gating mechanism, a dedicated sub-network that learns a per-voxel mixing coefficient to explicitly control the information flow from distinct pathways before they are fused.^15^ We hypothesized that a DL model equipped with a learned, spatially-varying gating mechanism could fuse image features with an optimized anatomical prior, leading to superior segmentation accuracy compared with rigid fusion methods, particularly for fine vasculature. Therefore, the purpose of this study was to develop and test a DL model that adaptively integrates an optimized anatomical prior to improve the accuracy of 3D hepatic vessel segmentation on CT, with a focus on fine, peripheral vasculature critical for surgical planning.

## Materials and methods

This retrospective study used de-identified public datasets only and, in accordance with local Institutional Review Board policy, was exempt from IRB review. Our method consists of 3 stages: (1) generation of a dataset-optimized anatomical prior map; (2) a DL network, PriM Fusion Net, that adaptively fuses information from the image and the prior to create an initial segmentation; and (3) a knowledge-based post-processing module to refine the segmentation and improve anatomical correctness. The intended use of the algorithm is as an add-on tool to automate the generation of 3D vascular models for preoperative surgical planning, substituting for manual segmentation. The target of the predictive model is a voxel-wise binary classification of hepatic vessels.

### Study cohort and data

We utilized the Medical Segmentation Decathlon (MSD) Task08-HepaticVessel dataset,^16^ which is comprised of 303 de-identified contrast-enhanced abdominal CT volumes acquired at Memorial Sloan Kettering Cancer Center (New York, NY) from patients with a variety of primary and metastatic liver tumors. No data were excluded from the original dataset. We used a refined version of the ground truth vessel labels provided by Xu et al.,^17^ which corrects known inconsistencies in the original annotations.

All CT volumes were resampled to a uniform isotropic spacing of 1.5 × 1.5 × 1.5 mm using third-order spline interpolation. A 5-fold cross-validation protocol was used for all experiments. For each fold, the dataset was partitioned at the patient level into training, validation, and testing sets with a 3:1:1 ratio. The partitioning was stratified to maintain an even distribution of thin-slice ([0.8, 2.0] mm) and thick-slice ([2.5, 8.0] mm) cases across all folds.

To assess cross-dataset generalizability, we additionally performed zero-shot external evaluation on the public 3D-IRCADb-01^18^ dataset, which contains CT scans from 20 patients (10 women and 10 men), with hepatic tumors present in 75% of cases.

### Anatomical prior generation

For each input 3D CT image (I), a binary liver mask was first obtained using the TotalSegmentator model.^19^ Within this mask, we generated a vessel-specific anatomical prior map ($P_{\text{frangi}\;}$) using the Frangi vesselness filter.^20^ The Frangi filter enhances tubular structures by analyzing the eigenvalues of the Hessian matrix at multiple scales. To ensure the prior was optimally suited for the dataset, we further tuned its parameters ($\left.\theta_{F}\right)\;$on the training set. The selected parameters were then frozen and applied unchanged to the corresponding validation and test sets. The detailed mathematical formulation and optimization of the filter are provided in the Supplemental Materials.

### PriM Fusion Net architecture

As illustrated in Figure 2, the PriM Fusion Net uses a dual-encoder architecture. The primary image pathway is a Swin Transformer-based encoder that processes the input image to extract features. In parallel, a lightweight 3D convolutional neural network (CNN) serves as the prior pathway, processing $P_{\text{frangi}\;}$ to extract prior features.

**Figure 2:**
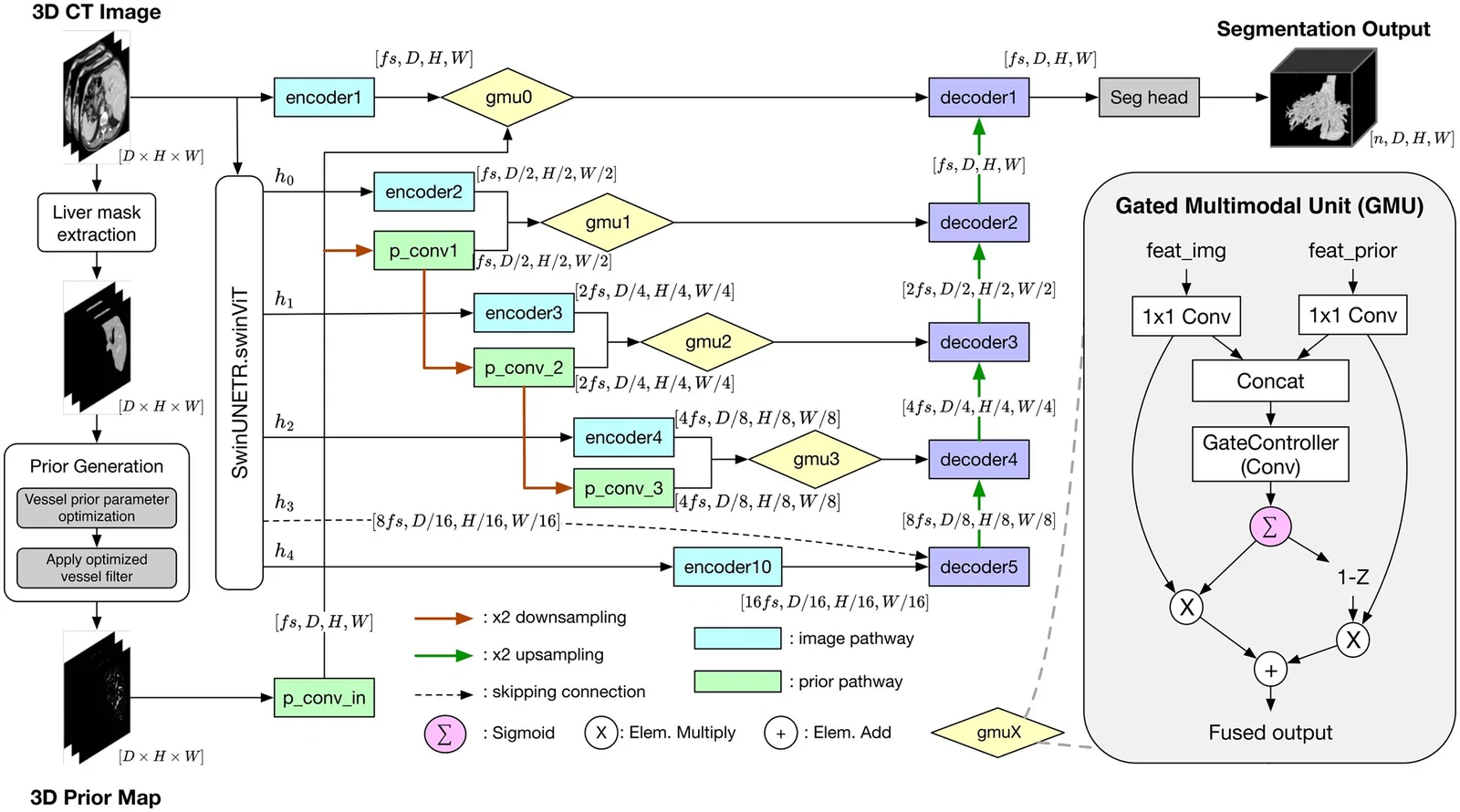
The input 3D image I undergoes optimized prior map generation, denoted as $P_{\mathrm{frangi}}$. The image III is then processed by a Swin Transformer-based encoder, while $P_{\mathrm{frangi}}$ is processed by a dedicated prior encoder. Features from corresponding levels of these 2 pathways are adaptively fused by GMUs, which serve as skip connections to the decoder. The decoder upsamples features from the image pathway’s bottleneck to produce the final vessel segmentation map $\hat{G}$. Abbreviation: GMU, gated multimodal unit.

A gated multimodal unit (GMU) adaptively fuses features from the image and prior pathways at 4 different scales by generating a spatially-varying gating map, $Z^{\left(s\right)}$, which produces a fused feature map, $F_{\text{fused}\;}^{\left(s\right)}$, by calculating a weighted average of the image features ($I_{f}^{\prime }$) and prior features ($P_{f}^{\prime }$) at every voxel:


(1) Ffused(s)=Z(s)⊙If′+(1-Z(s))⊙Pf′#

where ⊙ denotes element-wise multiplication. $F_{\mathrm{fused}}^{\left(s\right)}$ are passed via skip connections to a CNN-based decoder, which reconstructs the final binary segmentation map. Full details of the model architecture are provided in the Supplemental Materials.

### Model training and post-processing

The network was trained using a composite loss function combining Dice loss and Cross-Entropy loss. All models were implemented using the MONAI^21^ and PyTorch^22^ frameworks. Training was performed for 1800 epochs using the Adam optimizer with a cosine learning rate schedule. We used input patches of size 96×96×96 voxels and applied a comprehensive set of data augmentations, including random flips, rotations, and intensity shifts. The training was performed on a system equipped with 4 NVIDIA H100 GPUs, each with approximately 94 GB of memory.

The raw segmentation output from the network was refined using a rule-based pipeline implemented in the SimpleMind framework,^23^ as shown in Figure 3. The pipeline consists of a 3D morphological closing operation, connected component analysis, and a rule-based filtering step that removes components based on volume (<500 voxels) and anatomical location relative to the liver. The detailed specifications of SimpleMind post-processing are provided in the Supplemental Materials.

**Figure 3:**
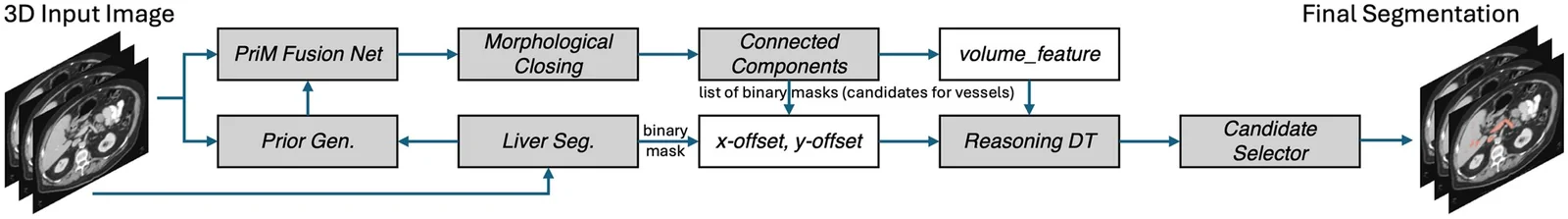
Rule-based post-processing flow in SimpleMind framework.

### Experimental design and statistical analysis

The primary outcome measures were the Dice similarity coefficient (DSC) and the 95th percentile Hausdorff distance (HD95). Performance was also stratified by vessel radius to assess the model’s efficacy on fine vasculature. Vessel radius was estimated for each ground truth voxel using a Euclidean Distance Transform, and voxels were grouped into 5 categories: ≤ 1.5 mm, 1.5-2.5 mm, 2.5-3.5 mm, 3.5-4.5 mm, and > 4.5 mm. These bins were chosen as pragmatic analytic strata to separate very small peripheral branches, intermediate branches, and larger central vessels while maintaining interpretable size groupings; they were not intended as formal clinical decision thresholds.

The mean and standard deviation for each performance metric were calculated across the 5 cross-validation folds. Two-sided paired t-tests were used to compare the performance of the proposed model against each baseline. To account for multiple comparisons, *P* values for the primary baseline comparisons were adjusted with the Holm-Bonferroni method; adjusted *P* < .05 was considered statistically significant.

### Model availability

The PriM Fusion Net implementation, including the trained model weights, post processing and related code used in this study, is available in the SimpleMind GitLab repository (https://gitlab.com/sm-ai-team/simplemind.git).

## Results

The experiments were conducted on the full cohort of 303 CT volumes, with performance metrics for each model calculated across the 5 test folds of the cross-validation. We compared PriM Fusion Net against 3 baselines: nnU-Net,^11^ a highly optimized U-Net framework; SwinUNETR, a representative Transformer-based model; and a multi-channel input model, which is a SwinUNETR architecture where a pre-computed vesselness filter output is concatenated with the original image as an additional input channel. We also evaluated PriM Fusion Net and SwinUNETR by zero-shot inference on the external 3DIRCADb dataset.

Dataset descriptors were limited by the public releases. For MSD Task08-HepaticVessel, detailed patient demographics, granular clinical variables, and full acquisition metadata (eg, scanner vendor/model, reconstruction kernel, and complete contrast-timing parameters) are not publicly available. For 3DIRCADb, limited public descriptors are available (20 patients; 10 women and 10 men; hepatic tumors in 75% of cases), but comprehensive clinical and acquisition metadata are likewise unavailable. Accordingly, these characteristics cannot be comprehensively reported for the evaluated cohorts.

### Quantitative analysis

The results of 5-fold cross-validation are presented in Table 1. PriM Fusion Net achieved a mean DSC of 77.68% ± 0.46% (95% confidence interval [CI]: 77.11, 78.25) and a mean HD95 of 11.55 ± 1.30 mm (95% CI, 9.94, 13.16), demonstrating a statistically significant improvement over all baseline models for both DSC and HD95 (all adjusted *P* < .02). The deep fusion strategy of PriM Fusion Net led to a 1.82% increase in DSC and a 31.5% reduction in HD95 compared to the multi-channel input model. Table 1 also details the computational complexity, showing that PriM Fusion Net contains 77.2M parameters, the highest among the tested models. Its computational load, measured at 581.8G floating-point operations (FLOPs), is higher than that of SwinUNETR (328.6G) due to its dual-encoder design but is lower than that of the convolution-based nnU-Net (743.3G). This indicates a trade-off in model complexity for higher accuracy.

**Table 1:** Five-fold cross-validation results comparing PriM Fusion Net with baseline models.

Method	Metric	Fold 1	Fold 2	Fold 3	Fold 4	Fold 5	Mean ± std [95% CI]	*P* value (vs Ours)	Params (M)^a^	FLOPs (G)^b^
nnU-Net	DSC (%)	70.25	71.42	68.37	73.02	71.49	70.91 ± 1.76 [68.73, 73.09]	<.02	**31.3**	743.3
	HD95 (mm)	25.8	27.28	22.18	16.96	23.28	23.10 ± 3.73 [18.47, 27.73]	<.02		
SwinUNETR	DSC (%)	75.47	74.96	73.52	75.92	74.13	74.80 ± 0.95 [73.62, 75.98]	<.02	62	**328.6**
	HD95 (mm)	18.56	19.19	19.25	14.67	25.17	19.37 ± 3.65 [14.84, 23.90]	<.02		
Multi-Channel Input	DSC (%)	75.72	75.18	75.88	76.36	76.17	75.86 ± 0.44 [75.31, 76.41]	<.02	62	**328.6**
	HD95 (mm)	17.69	16.94	17.9	13.39	18.36	16.86 ± 1.94 [14.45, 19.27]	<.02		
PriM Fusion Net (Ours)	DSC (%)	77.32	77.38	77.26	78.28	78.15	**77.68 ± 0.46 [77.11, 78.25]**	—	77.2	581.8
	HD95 (mm)	13.57	10.46	12.05	11.2	10.47	**11.55 ± 1.30 [9.94, 13.16]**	—		

Values are mean ± standard deviation, with 95% confidence intervals in brackets. *P* values compare each baseline with PriM Fusion Net after Holm-Bonferroni adjustment. Bold indicates the best value within each metric row.

a Params (M): model parameters in millions.

b FLOPs (G): Giga Floating Point Operations, calculated for a 96 × 96 × 96 input patch.

Abbreviations: DSC, Dice similarity coefficient; FLOPs, floating-point operations; HD95, 95th percentile Hausdorff distance; Params, model parameters.

### Qualitative results

Figure 4 shows a comparison of 2 challenging cases from the test set. In the first case (top row), both nnU-Net and SwinUNETR fail to delineate fine, bifurcating vascular branches (orange box). The multi-channel input model detects portions of these branches but yields a disconnected segmentation. PriM Fusion Net delineates these branches with improved connectivity, while the SimpleMind post-processing module removes extraneous, isolated components. The second case (bottom row) involves a region with dense, fine vessels (blue box). Here, SwinUNETR shows a marginal improvement over nnU-Net, and the multi-channel model segments more of the small vessels. PriM Fusion Net produces the most accurate result, capturing both the quantity and the continuity of these structures. A particularly thin vessel branch (orange box), largely missed by all baseline methods, was identified by our network. The main residual failure modes were very thick-slice CT scans with severe partial-volume effects and highly heterogeneous parenchymal texture, where the finest peripheral vessels could still be missed or small false positives could appear.

**Figure 4:**
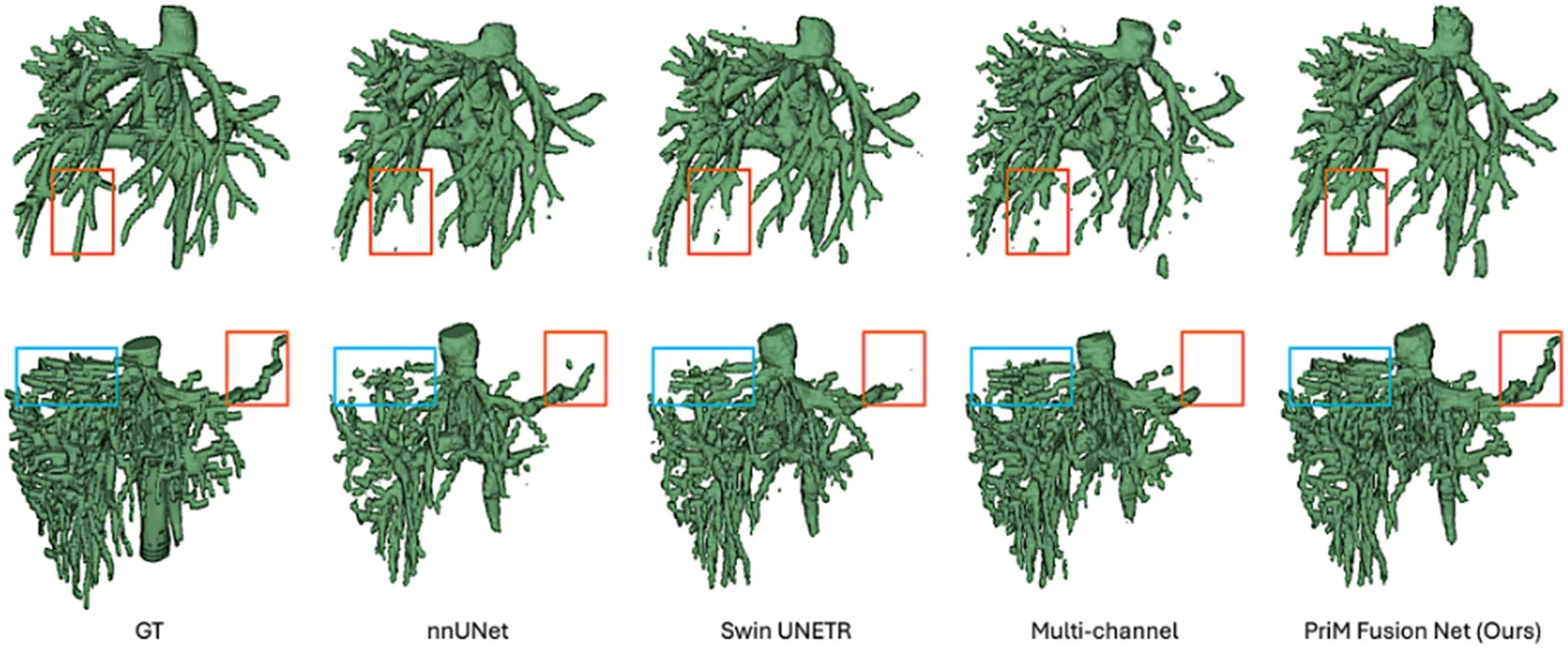
Visual comparison of segmentation results on 2 challenging cases from the test set. Each row represents a case and shows (from left to right) Ground Truth (GT), nnU-Net, SwinUNETR, Multi-channel Input, and PriM Fusion Net. Highlighted regions mark fine bifurcating or very thin peripheral branches that are incompletely recovered by the baseline models and a dense cluster of fine vessels where the methods differ in completeness and continuity.

### Ablation analysis

We conducted an ablation study with results summarized in Table 2. The baseline SwinUNETR architecture achieved a mean DSC of 74.80% and HD95 of 19.37 mm. Concatenating a generic, non-optimized Frangi prior to the input slightly degraded the performance (DSC 74.59%). Optimizing the prior before concatenation provided a clear benefit, improving the DSC to 75.86% and reducing the HD95 to 16.86 mm. However, the most significant performance gain was observed when replacing rigid concatenation with the adaptive GMUs, which increased the DSC by 1.79% to 77.65% and reduced the HD95 to 14.98 mm, confirming the superiority of the learned, deep fusion mechanism. Finally, the addition of the SimpleMind post-processing module provided a modest increase in DSC but substantially improved the HD95, highlighting its effectiveness at refining the segmentation boundary and improving anatomical plausibility. A qualitative visualization of this refinement is shown in Figure 5.

**Figure 5:**
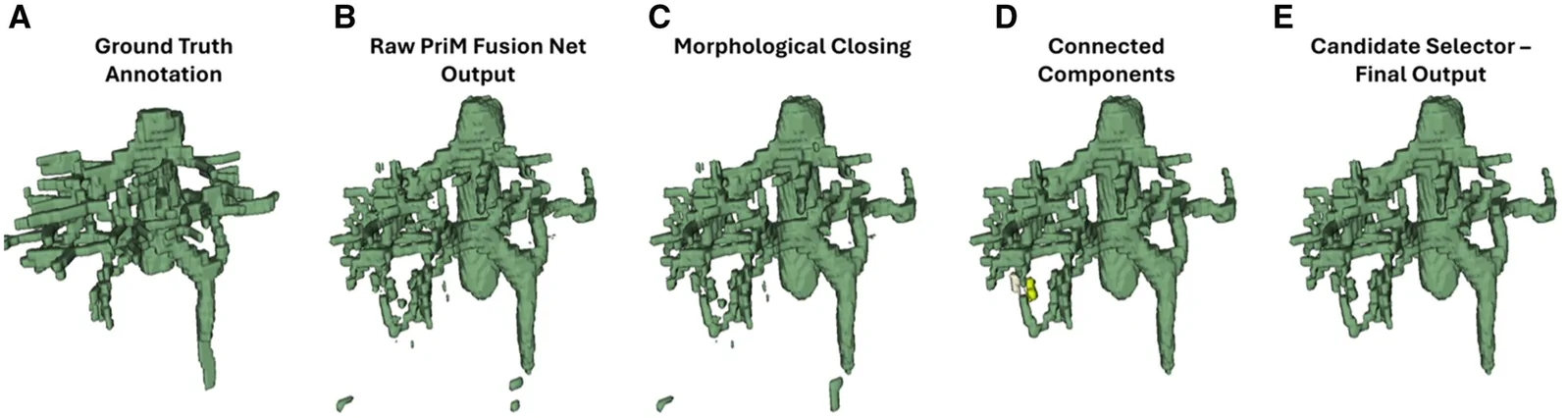
Visual illustration of the SimpleMind post-processing pipeline on a representative test case. From left to right: (A) 3D Ground Truth annotation; (B) raw output from PriM Fusion Net (DSC = 0.7662, HD = 299.33 mm); (C) result after morphological closing to bridge minor gaps; (D) identification of all connected components as candidates; (E) final refined segmentation after the Candidate Selector (DSC = 0.7952, HD = 26.54 mm). Abbreviations: DSC, Dice similarity coefficient; HD, Hausdorff distance.

**Table 2:** Ablation study of PriM Fusion Net components.

Configuration	DSC (%) mean ± std [95% CI]	HD95 (mm) mean ± std [95% CI]
Baseline (SwinUNETR)	74.80 ± 0.95 [73.62, 75.98]	19.37 ± 3.65 [14.84, 23.90]
+ Prior (Generic) + Concat	74.59 ± 1.05 [73.29, 75.89]	19.81 ± 3.98 [14.87, 24.75]
+ Prior (Optimized) + Concat	75.86 ± 0.44 [75.31, 76.41]	16.86 ± 1.94 [14.45, 19.27]
+ Prior (Optimized) + GMU	77.65 ± 0.59 [76.92, 78.38]	14.98 ± 3.28 [10.91, 19.05]
PriM Fusion Net (Full)	**77.68 ± 0.46 [77.11, 78.25]**	**11.55 ± 1.30 [9.94, 13.16]**

Values are mean ± standard deviation, with 95% confidence intervals in brackets. Bold indicates the best-performing configuration for each metric.

Abbreviations: DSC, Dice similarity coefficient; GMU, gated multimodal unit; HD95, 95th percentile Hausdorff distance.

### Analysis of small vessel and gating mechanisms

To evaluate the model’s performance on the most clinically challenging structures, we stratified the ground truth annotations into 5 categories based on local vessel radius (Figure 6A). The comparative results, shown in Table 3, reveal that while PriM Fusion Net and the baseline SwinUNETR perform comparably on large vessels (radius > 3.5 mm), a significant performance gap emerges as vessel size decreases. For the smallest vessel category (radius ≤ 1.5 mm), PriM Fusion Net achieved a DSC of 59.2%, a 13.5% improvement over the SwinUNETR baseline (45.7%). This indicates that the adaptive prior-fusion strategy is particularly effective for recovering fine vascular structures.

**Figure 6:**
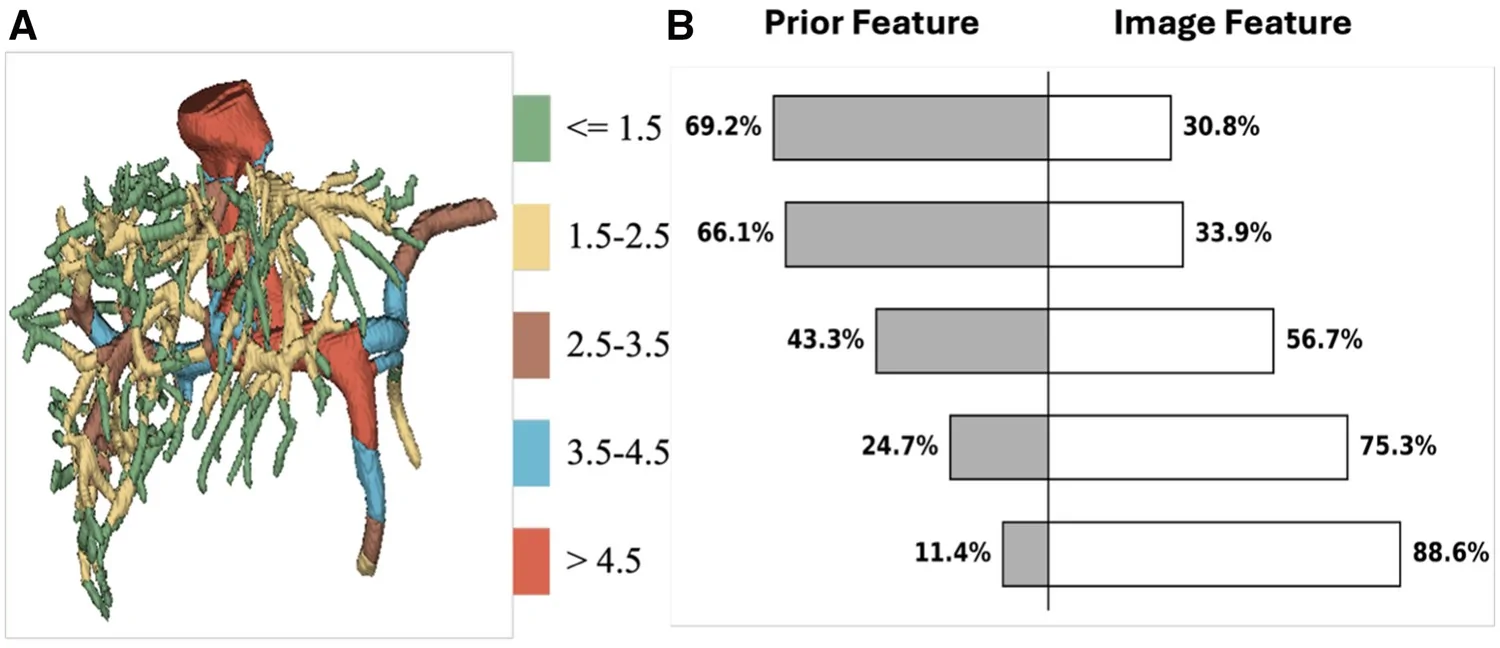
Quantitative analysis of the gated multimodal unit (GMU). (A) Visualization of the 5 vessel size categories derived from the ground truth segmentation, with colors representing vessel radius: > 4.5 mm (red), 3.5-4.5 mm (blue), 2.5-3.5 mm (brown), 1.5-2.5 mm (yellow), and ≤ 1.5 mm (green). (B) Diverging bar plot of the mean learned contribution weights (%) of image features versus prior features for voxels in each size category on the testing set. The pattern indicates greater reliance on image features for large vessels and greater reliance on prior features for smaller, more challenging vessels.

**Table 3:** Size stratification with DSC and sensitivity.

Vessel radius (mm)	SwinUNETR DSC (%)	SwinUNETR sensitivity (%)	PriM Fusion Net DSC (%)	PriM Fusion Net sensitivity (%)
> 4.5	88.7 ± 1.5 [86.84, 90.56]	90.2 ± 1.8 [87.97, 92.43]	**88.8 ± 1.3 [87.19, 90.41]**	**90.5 ± 1.5 [88.64, 92.36]**
3.5-4.5	**84.3 ± 2.1 [81.69, 86.91]**	85.1 ± 2.4 [82.12, 88.08]	**84.3 ± 1.9 [81.94, 86.66]**	**85.5 ± 2.0 [83.02, 87.98]**
2.5-3.5	78.6 ± 2.8 [75.12, 82.08]	77.4 ± 3.2 [73.43, 81.37]	**82.1 ± 2.5 [79.00, 85.20]**	**81.6 ± 2.6 [78.37, 84.83]**
1.5-2.5	69.2 ± 3.5 [64.86, 73.54]	65.8 ± 4.1 [60.71, 70.89]	**76.8 ± 3.1 [72.95, 80.65]**	**74.2 ± 3.4 [69.98, 78.42]**
≤ 1.5	45.7 ± 4.2 [40.49, 50.91]	42.3 ± 5.5 [35.47, 49.13]	**59.2 ± 3.8 [54.48, 63.92]**	**56.4 ± 4.2 [51.19, 61.61]**

Performance reported as mean ± std across the 5-folds. Values are mean ± standard deviation across the 5-folds, with 95% confidence intervals in brackets. Bold indicates the better-performing model within each vessel-radius stratum and metric. Sensitivity denotes recall within the specified vessel-radius stratum.

Abbreviation: DSC, Dice similarity coefficient.

The adaptive behavior of the GMU was analyzed to explain this performance differential. We focused on the gating signal$Z^{\left(0\right)}$ from the GMU at the highest resolution (s = 0 from gmu0 in Figure 2 and eqn (1)), where $Z^{\left(0\right)}\to 1$ indicates reliance on image features and $Z^{\left(0\right)}\to 0$ indicates reliance on prior features. For our analysis, at a voxel level, $Z^{\left(0\right)}>0.5$ is interpreted as an overall emphasis on the image pathway, while $Z^{\left(0\right)}\leq 0.5$ indicates a greater reliance on the prior. Figure 6B shows the average gating signal value for voxels within each vessel size category on the testing set. For the largest vessels (>4.5 mm), which are well-defined in CT scans, the model primarily relies on Swin Transformer-derived image features, assigning them an average contribution weight of 88.6%. As vessel size decreases and the CT signal becomes less distinct, the model’s reliance shifts toward the prior. For the smallest and most challenging vessels (radius ≤ 1.5 mm), the model learns to trust the Frangi prior features most, assigning them a dominant contribution weight of 69.2%. This quantitative analysis of the gating signal provides a direct explanation for the performance improvements observed in small (radius ≤ 3.5 mm) vessel segmentation (Table 3). The network learns to dynamically balance between information sources, trusting the high-fidelity image features for large, distinct structures and shifting its reliance to the engineered prior where the image signal is ambiguous.

### External validation

To assess generalizability, we performed zero-shot inference on the external 3DIRCADb dataset (Table 4). Under this domain shift, SwinUNETR achieved a mean DSC of 65.65% ± 6.28% (95% CI, 62.71, 68.59) and HD95 of 13.26 ± 7.93 mm (95% CI, 9.55, 16.97). PriM Fusion Net remained superior, with a DSC of 68.42% ± 5.56% (95% CI, 65.82, 71.02) and HD95 of 11.78 ± 7.01 mm (95% CI, 8.50, 15.06). These findings suggest that the prior-modulated fusion strategy retained value on an unseen external cohort without fine-tuning.

**Table 4:** External validation results on the 3DIRCADb dataset (zero-shot inference).

Method	DSC (%) mean ± std [95% CI]	HD95 (mm) mean ± std [95% CI]	Sensitivity (%) mean ± std [95% CI] for ≤ 1.5 mm
SwinUNETR (Baseline)	65.65 ± 6.28 [62.71, 68.59]	13.26 ± 7.93 [9.55, 16.97]	51.45 ± 6.35 [48.48, 54.42]
PriM Fusion Net (Ours)	**68.42 ± 5.56 [65.82, 71.02]**	**11.78 ± 7.01 [8.50, 15.06]**	**55.18 ± 5.19 [52.75, 57.61]**

Values are mean ± standard deviation, with 95% confidence intervals in brackets. Bold indicates the better model for each metric.

Abbreviations: DSC, Dice similarity coefficient; HD95, 95th percentile Hausdorff distance.

## Discussion

In this study, we developed and tested PriM Fusion Net, a DL framework that adaptively integrates an optimized anatomical prior to improve 3D hepatic vessel segmentation. Our results showed that this approach yields statistically significant improvements in segmentation accuracy and anatomical correctness over strong baselines, including a standard rigid-fusion method. The principal finding is that the model’s adaptive fusion mechanism is particularly effective at delineating fine, peripheral vasculature, addressing a key limitation of current automated methods.

From a technical standpoint, improved fine-vessel recovery may improve the completeness and spatial fidelity of the vascular tree used for downstream hepatic treatment planning. Portal and hepatic venous anatomy underlies Couinaud segment boundaries and may influence future liver remnant estimation, segment-oriented resection design, and selective embolization planning. However, the present study evaluated segmentation accuracy rather than any treatment task, so these potential uses should be interpreted as hypotheses rather than demonstrated clinical benefit.

A plausible downstream workflow is preoperative 3D model generation for hepatobiliary surgeons, interventional radiologists, and 3D-modeling specialists reviewing portal-venous phase CT before liver resection, ablation, or embolization. In that setting, the model output would serve as an automatically generated first-pass portal/hepatic venous segmentation that can be reviewed and corrected before vascular visualization or territory-based planning. The intermediate workflow benefit would be less manual contouring and a lower risk of omitting peripheral venous branches during model preparation. If validated for generalizability in larger, more heterogeneous cohorts and effectively deployed in a clinical practice environment, this could improve the fidelity of treatment planning, preserve venous drainage, or avoid unintended vessel injury. These workflow effects and any patient-level benefit were not directly measured in this study.

Our study provides a key insight: the method of prior integration is as critical as the prior itself. Simply concatenating a generic vesselness filter degraded performance. This rigid approach is ill-suited for the hepatic vasculature, which has a wide spectrum of vessel calibers. Research by Garret et al.,^12^ for example, demonstrated the trade-off of this method: while rigid fusion improved small vessel detection, it simultaneously degraded performance for larger vessels.

Our deep, adaptive GMUs were designed to solve this specific trade-off. By replacing rigid concatenation with a learned, spatially varying gating mechanism, the model can learn a dynamic policy. The gating-signal analysis suggests that the network relies more on image features for large, distinct vessels and shifts toward the engineered prior where the image signal is ambiguous. At the same time, our evaluation remained limited to DSC and HD95. Although useful for overlap and boundary error, these metrics do not fully capture vascular branching topology, continuity, or connectivity, which are important for surgical and interventional planning. Future work should therefore incorporate explicit topology or continuity metrics in addition to clinician-relevant workflow endpoints.

This study has several limitations. First, no clinically relevant downstream task, planning study, or patient-outcome study was performed. So, whether the observed segmentation gains improve Couinaud classification, treatment planning, or clinical outcomes remains unknown. Second, the main development dataset was a single public portal-venous phase cohort, and the Frangi prior was optimized on that dataset, so overfitting to acquisition characteristics remains a concern despite the external zero-shot results. Moreover, the public datasets do not provide comprehensive demographic, clinical, or acquisition metadata; for 3DIRCADb, only limited descriptors are available. Consequently, the specific patient populations, scanners, protocols, and clinical use cases to which the model generalizes remain incompletely defined. Third, the target anatomy was limited to portal and hepatic veins; the method did not address hepatic arteries, which are much less conspicuous on portal-venous phase CT. Fourth, our experimental design was structured to evaluate the proposed fusion methodology rather than to provide an exhaustive benchmark against all contemporary vessel segmentation networks. Future work should benchmark the model against other domain-specific methods such as VSNet 18. Finally, our model’s design prioritized segmentation accuracy over computational efficiency, a deliberate trade-off made to address the inherent difficulty of the task. As performance approaches clinical acceptability, future research could investigate model compression techniques or more parameter-efficient backbones such as UNETR++^24^ or foundational models such as VesselFM^25^ to reduce the model’s footprint for practical deployment.

In conclusion, PriM Fusion Net demonstrates that deep, adaptive fusion of CT image features with an optimized anatomical prior can improve the segmentation of portal and hepatic veins, especially in fine peripheral branches that challenge standard models. The approach improved both internal and external performance relative to strong baselines and generated more anatomically plausible segmentations after post-processing. These findings support further evaluation of prior-modulated fusion as a component of 3D vascular modeling pipelines for liver surgery and intervention.

## Supplementary Material

umag038_Supplementary_Data

## Data Availability

Imaging data are publicly available from the Medical Segmentation Decathlon Task 08 (Hepatic Vessel) dataset (de-identified). Refined vessel labels used for evaluation follow the public release described by Xu et al. Source code and trained model weights are available in the SimpleMind GitLab repository (https://gitlab.com/sm-ai-team/simplemind.git).
