# Supplementary material for: Adaptive deep learning model with anatomical priors for 3D segmentation of fine hepatic vasculature on CT
Source: Radiol Adv. 2026 Nov 3;3(6):umag038. doi: 10.1093/radadv/umag038 (PMC13769431; doi:10.1093/radadv/umag038)
Supplement: Supplementary file 1 [file umag038_supplementary_data.zip › Supplementary_material.zip!/RADADV-2026-064_supplementary_material.pdf]

Adaptive Deep Learning Model with Anatomical Priors for 3D Segmentation of Fine Hepatic Vasculature on CT

Zixuan Zhao[1,2], MS; Kalyani Vyapari[2], MS; Kyunghyun Sung[1,2], PhD; David S. Lu[2], MD; Grace H. Kim[1,2], PhD; and Matthew S. Brown[1,2], PhD

[1]Physics and Biology in Medicine Graduate Program, David Geffen School of Medicine at UCLA, Los Angeles, California, USA

[2]Department of Radiological Sciences, David Geffen School of Medicine at UCLA, Los Angeles, California, USA

Address correspondence to:

Zixuan Zhao, MS

Center for Computer Vision and Imaging Biomarkers

Department of Radiological Sciences

David Geffen School of Medicine at UCLA

924 Westwood Blvd, Suite 615, Los Angeles, CA 90024

Phone: 850.888.0514; Email: zixuanzhao@mednet.ucla.edu

Supplementary Material

Supplementary Material: Detailed Formulations

Frangi Vesselness Filter Formulation and Optimization

The flowshart for the prior map optimization is shown in Figure S1. The Frangi filter enhances tubular structures by analyzing the eigenvalues of the Hessian matrix. For a voxel at a given scale , the Hessian matrix is computed. Its eigenvalues are denoted by and sorted by magnitude such that . For an ideal bright vessel on a dark background, the eigenvalues should satisfy , with and being large negative numbers of similar magnitude.$\sigma\mathcal{H}(\sigma)\lambda_1, \lambda_2, \lambda_3|\lambda_1| \leq |\lambda_2| \leq |\lambda_3|\lambda_1 \approx 0\lambda_2\lambda_3$

The vesselness measure at scale $\sigma, V_o(\sigma)$, is defined as:

$$V_o(\sigma) = \begin{cases} 0, & \text{if } \lambda_2 > 0 \text{ or } \lambda_3 > 0 \\ \left(1 - \exp\left(-\frac{R_A^2}{2\alpha^2}\right)\right) \cdot \exp\left(-\frac{R_B^2}{2\beta^2}\right) \cdot \left(1 - \exp\left(-\frac{S^2}{2\gamma^2}\right)\right), & \text{otherwise} \end{cases}$$

where the terms R_A, R_B, and S are defined as:

$$R_A = \frac{|\lambda_2|}{|\lambda_3|},\ R_B = \frac{|\lambda_1|}{\sqrt{|\lambda_2 \lambda_3|}},\ S = \sqrt{\lambda_1^2 + \lambda_2^2 + \lambda_3^2}$$

The parameters α, β, γ control the filter's sensitivity to deviations from an ideal tubular shape, the discrimination between line-like and blob-like structures, and the suppression of unstructured noise, respectively. The final Frangi map P_{frangi} is obtained by taking the maximum response across all evaluated scales in a predefined interval $[\sigma_{\text{min}}, \sigma_{\text{max}}]$.

To ensure the prior was optimally suited for the dataset, the Frangi filter's parameters (θ_F) were tuned by maximizing the Area Under the Precision-Recall Curve

(AUC-PR) against the ground truth vessel masks on the training set. The optimal parameter set, θ_F^*, was determined as:

$$\theta_F^* = \arg\max_{\theta_F} \frac{1}{N_{tr}} \sum_{k=1}^{N_{tr}} \text{AUC-PR}\left(P_{\text{frangi}}\left(I_k, \theta_F\right), G_k\right)$$

where N_{tr} is the number of images in the training set, I_k is the k-th training image, G_k is its corresponding ground truth mask, and θ_F represents the set of Frangi filter parameters. This optimized parameter set was then fixed and used to generate prior maps for all images.

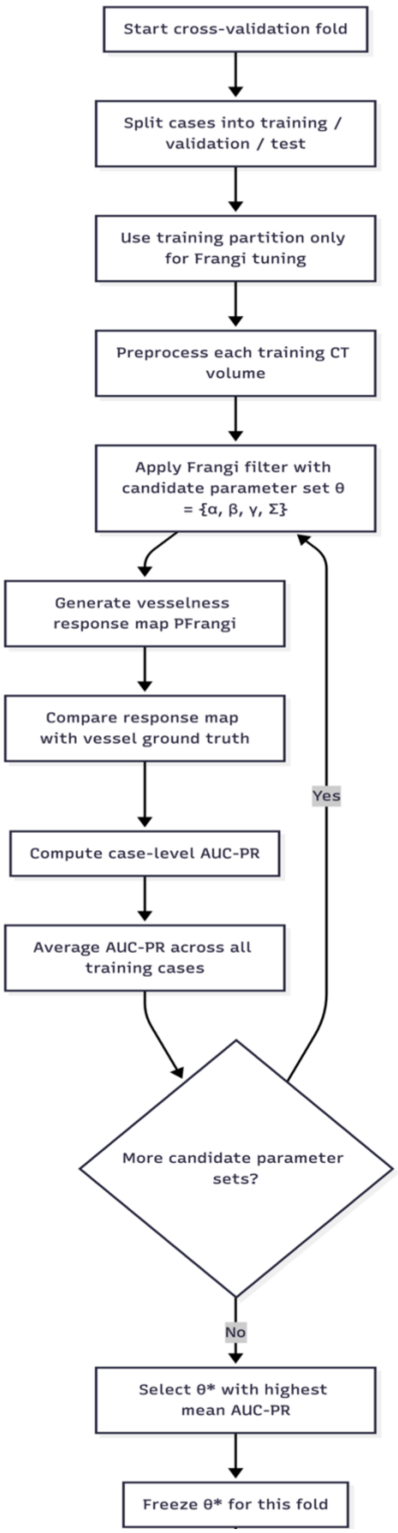

Figure S1. Flowchart for prior map optimization.

Detailed Network Formulations

Image Pathway Encoder

The image pathway processes the normalized image $I_{\text{norm}} \in \mathbb{R}^{1\times D\times H\times W}$. An initial convolutional block produces low-level features:

$$I_{enc}^{(0)} \in \mathbb{R}^{C_0\times D\times H\times W}$$

The core Swin Transformer backbone applies a sequence of stages (S_0, S_1, S_2, S_3) to produce hierarchical feature maps:

$$h_j \in \mathbb{R}^{C_j'\times D_j\times H_j\times W_j},\ j = 0,1,2,3$$

Each h_j captures hierarchical features at a downsampling factor of 2^j relative to the input. We apply convolutional blocks $E_{\text{conv}}^{(j)}$ (with the same structure as $E_{\text{conv}}^{(0)}$) to map:

$$I_{enc}^{(1)} = E_{conv}^{(1)}(h_0),\ I_{enc}^{(2)} = E_{conv}^{(2)}(h_1),\ I_{enc}^{(3)} = E_{conv}^{(3)}(h_2).$$

Channel dimensions are set as $C_1 = fs, C_2 = 2fs, C_3 = 4fs$. The deepest Swin output

$$h_3 \in \mathbb{R}^{C\,'_3\times D_3\times H_3\times W_3}$$

is processed by a convolutional block $E_{conv}^{(BN)}$ to produce the bottleneck feature

$$I_{BN} \in \mathbb{R}^{C_{BN}\times D_3\times H_3\times W_3},\ C_{BN} = 16\text{fs}$$

I_{BN} is the initial input to the decoder.

Prior Encoder Pathway

The prior pathway is a lightweight 3D CNN that extracts multi-scale features from the normalized prior map $P_{\text{norm}} \in \mathbb{R}^{1\times D\times H\times W}$. Its feature scales match those of $I_{\text{enc}}^{(s)}$ for fusion. Denote $P_{\text{enc}}^{(s)}$ as the prior feature at scale s :

- $P_{\text{enc}}^{(0)} \in \mathbb{R}^{C_0\times D\times H\times W}$ is produced by $E_{\text{prior}}^{(0)}$ acting on P_{norm} .
- For $s = 1,2,3, P_{\text{enc}}^{(s)}$ is obtained by downsampling via strided 3D convolution followed by $E_{\text{prior}}^{(s)}$. Each $E_{\text{prior}}^{(s)}$ comprises two 3D convolutions, normalization, and activation, yielding channel dimension C_s and resolution $D_s \times H_s \times W_s$ that align with $I_{\text{enc}}^{(s)}$. Specifically, $C_1 = fs$, $C_2 = 2fs, C_3 = 4fs$.

Gated Multimodal Unit (GMU)

At each scale $s \in \{0,1,2,3\}$, a Gated Multimodal Unit $GMU^{(s)}$ fuses image features $I_{enc}^{(s)}$ and prior features $P_{\text{enc}}^{(s)}$ into a single feature map $F_{\text{fused}}^{(s)}$ of dimension $C_s \times D_s \times H_s \times W_s$. Denote $I_f = I_{\text{enc}}^{(s)}$ and $P_f = P_{enc}^{(s)}$ with both having C_s channels. The fusion proceeds as:

- Feature Projection: Project I_f and P_f via $1 \times 1 \times 1$ convolutions to match dimensions:

  $$I_f' = \text{Conv}_{1\times1\times1}(I_f)\,,\ P_f' = \text{Conv}_{1\times1\times1}(P_f).$$

  If C_s already matches, set $I_f' = I_f, P_f' = P_f$.

- Gate Generation: Concatenate I_f' and P_f' along the channel axis:

  $$X_{cat} \in \mathbb{R}^{2Cs\times D_s\times H_s\times W_s}.$$

Pass X_{cat} through a gate controller $GC^{(s)}$, a shallow CNN (two 3D convolutions followed by sigmoid), to produce a gating map:

$$Z^{(s)} = \sigma\left(GC^{(s)}(X_{\text{cat}})\right),\ Z^{(s)} \in [0,1]^{Cs \times D_s \times H_s \times W_s}.$$

- Gated Fusion: Compute fused output:

$$F_{fused}^{(s)} = Z^{(s)} \odot I_f' + \left(1 - Z^{(s)}\right) \odot P_f'$$

where $\odot$ denotes element-wise multiplication.

Decoder Pathway

The decoder reconstructs the segmentation map by recursively upsampling features and integrating the fused skip connections from the GMUs. The process begins at the deepest stage:

$$D_{out}^{(4)} = D_{up}^{(4)}(I_{BN}, h_3)$$

And proceeds for subsequent stages $s = 3,2,1,0$:

$$D_{\text{out}}^{(s)} = D_{\text{up}}^{(s)}\left(D_{\text{out}}^{(s+1)}, F_{\text{fused}}^{(s+1)}\right)$$

Finally, a full-resolution feature map is obtained:

$$F_{\text{final}} = D_{\text{up}}^{(-1)}\left(D_{\text{out}}^{(0)}, F_{\text{fused}}^{(0)}\right)$$

The final output layer applies a $1 \times 1 \times 1$ convolution to F_{final} to produce the segmentation logits $\hat{L}$.

Loss Function and Data Augmentation

The network was trained using a composite loss function combining Dice loss and Cross-Entropy loss ($\mathcal{L}_{\text{DiceCE}}$), which is formulated as:

$$\mathcal{L}_{\text{DiceCE}} = \mathcal{L}_{\text{Dice}}\left(\text{softmax}(\hat{L}), G\right) + \mathcal{L}_{CE}\left(\text{softmax}(\hat{L}), G\right)$$

The Dice loss for the foreground vessel class ($c = 1$) is formulated as:

$$\mathcal{L}_{\text{Dice}}^{(c=1)} = 1 - \frac{2\sum_i \hat{p}_{ic} G_{ic} + \epsilon}{\sum_i \hat{p}_{ic}^2 + \sum_i G_{ic}^2 + \epsilon}$$

where $\hat{p}_{ic}$ is the predicted probability for voxel i belonging to class c and G_{ic} is the corresponding ground truth label. The Cross-Entropy loss is given by:

$$\mathcal{L}_{CE} = -\sum_i \sum_c G_{ic} \log(\hat{p}_{ic})$$

During training, 3D patches were augmented online using stochastic spatial and intensity transformations. Spatial augmentation included random axis flips and random rotations of the volumetric patch. Intensity augmentation included random intensity perturbations applied after image normalization. All augmentations were applied only to training patches and were disabled for validation, testing, and external evaluation.

Data Augmentation Specification

To improve robustness to orientation changes, local appearance variation, and scanner-dependent intensity differences, we applied stochastic augmentation during training including Random spatial flips, Random rotations and Intensity-based transformations. Random flips were applied along anatomical axes with stochastic triggering during training. Random rotations were applied to expose the model to modest orientation variability not explicitly represented in the original dataset.

These augmentations were applied to the input CT volume and corresponding label map in a synchronized manner for geometry-preserving operations, so that spatial consistency between image and segmentation label was maintained.

SimpleMind Postprocessing Specification

To improve anatomical plausibility and reduce surface outliers, the raw network output was refined using a rule-based post-processing module implemented in the **SimpleMind** framework. This stage was applied after voxelwise inference and was designed to suppress isolated false positives and anatomically implausible disconnected fragments.

Input to post-processing

The segmentation network produced a voxelwise vessel probability map. This probability map was converted to a binary candidate mask using a **threshold of 0.5**:

$$\hat{G}_{\text{bin}}(x) = \begin{cases} 1, & \text{if } p(x) \geq 0.5, \\ 0, & \text{otherwise.} \end{cases}$$

This thresholded mask served as the input to the rule-based refinement stage.

Morphological consolidation

To reduce small internal gaps and improve local continuity before component analysis, the binary mask underwent **morphological closing** using a **spherical structuring element with radius 2 voxels**. This operation was used to connect immediately adjacent vessel fragments separated by tiny discontinuities while avoiding aggressive over-smoothing.

Connected-component extraction

After morphological closing, the mask was decomposed into its connected components. Each connected component was treated as a candidate vascular structure. For every candidate, the following features were extracted:

- **component volume** (number of voxels),
- **component centroid** in image coordinates.

These component-level features were then passed to the rule-based filtering stage.

Anatomical rule-based filtering

SimpleMind then evaluated each connected component using explicit anatomical heuristics. Two classes of criteria were applied:

(a) Volume constraint

Candidates with volumes outside the allowed range $[500, 10^6]$ voxels were rejected. This rule was intended to suppress tiny isolated false-positive fragments while preserving large connected vascular structures.

(b) Spatial consistency constraint

For spatial validation, the centroid of each candidate component was compared with the centroid of a reference liver mask. Components were rejected if their centroid offset from the liver centroid exceeded ± 15**cm** in the in-plane spatial directions used by the rule engine. This rule was intended to eliminate disconnected candidates located implausibly far from the liver region.

Final candidate selection

Components that satisfied both the volume and spatial consistency criteria were retained and merged to form the final refined vessel mask:

$$\tilde{G} = \bigcup_{c \in \mathcal{C}_{\text{valid}}} c,$$

where $\mathcal{C}_{\text{valid}}$denotes the set of candidate components surviving rule-based filtering.
